# Supplementary material for: Identification of photoperiod-induced specific miRNAs in the adrenal glands of Sunite sheep (Ovis aries)
Source: Front Vet Sci. 2022 Jul 22;9:888207. doi: 10.3389/fvets.2022.888207 (PMC9354845; doi:10.3389/fvets.2022.888207)
Supplement: Supplementary file 1 [file Data_Sheet_1.ZIP › Supplementary file/miRDeep2 file.zip!/miRDeep2 file/Know_Structure/LP42A1/oar-mir-29a.pdf]

miRBase precursor : oar-mir-29a
Total read count : 143140
oar-miR-29a read count : 143109
remaining reads : 31

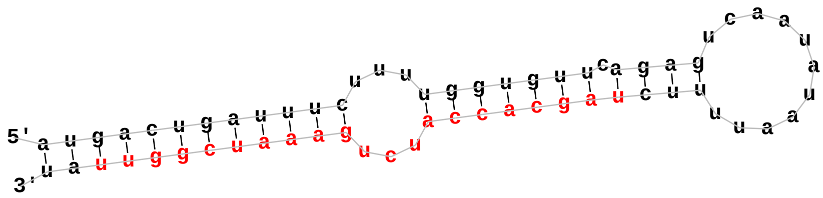

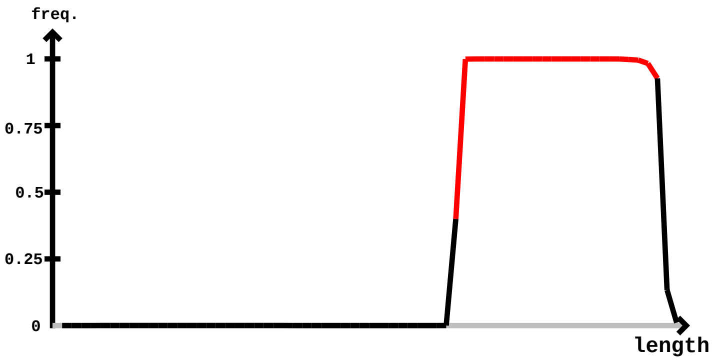

oar-miR-29a

```
5'- augacugauuucuuuugguguucagagucaauauaauuuucuagcaccaucugaaaucgguuau  -3'   exp
    ((((((((((((...(((((((.((((...........)))))))))))...))))))))))))    reads    mm    sample
    .ugacugauuucuuuugguguu..........................................    2        0     seq
    .ugacugauuucuuuugguguuc.........................................    3        0     seq
    ...acugauuucuuuuggugGu..........................................    1        1     seq
    ...acugauuucuuuugguguu..........................................    1        0     seq
    ...acugauuucuuuugguguuc.........................................    21       0     seq
    ...........................ucaauauaauuuucuagcaccauc.............    1        0     seq
    ......................................uucuagcaccaucugaaaucgg....    1        0     seq
    ......................................uucuagcaccaucugaaaucggu...    1        0     seq
    .......................................ucuagcaccaucugaaauc......    2        0     seq
    .......................................ucuagcaccaucugaaaucgg....    4        0     seq
    .......................................ucuagcaccauUugaaaucgg....    1        1     seq
    .......................................ucuagcaccaucugaaaucggu...    11       0     seq
    .......................................ucuagcaccaucugaaaucgguu..    9        0     seq
    ........................................cuagcaccaucugaaau.......    7        0     seq
    ........................................cuagcaccaucugaaaA.......    2        1     seq
    ........................................cuagcaccaucugaaauA......    4        1     seq
    ........................................cuagcaccaucugaaauc......    77       0     seq
    ........................................cuagcaccaucugaaaucg.....    79       0     seq
    ........................................cuagcaccaucugaaaucgg....    281      0     seq
    ........................................cuagcaccauUugaaaucgg....    1        1     seq
    ........................................cuagcaccaucugaaaucggu...    2294     0     seq
    ........................................cuagcaccauUugaaaucggu...    31       1     seq
    ........................................cuagcaccauUugaaaucgguu..    212      1     seq
    ........................................cuagcaccaucugaaaucgguu..    52954    0     seq
    ........................................cuagcaccauUugaaaucgguua.    3        1     seq
    ........................................cuagcaccaucugaaaucgguua.    1171     0     seq
    ........................................cuagcaccauUugaaaucgguuau    1        1     seq
    ........................................cuagcaccaucugaaaucgguuau    95       0     seq
    .........................................uaUcaccaucugaaauc......    1        1     seq
    .........................................uagcaccaCcugaaauc......    1        1     seq
    .........................................uagcaccaucugaaauU......    25       1     seq
    .........................................uagcaccaucugaaauA......    7        1     seq
    .........................................uagcaccaucugaaauc......    168      0     seq
    .........................................uGgcaccaucugaaauc......    2        1     seq
    .........................................uagcaccaucugaaGuc......    1        1     seq
```

oar-miR-29a

```
augacugauuucuuuugguguucagagucaauauaauuuucuagcaccaucugaaaucgguuau
.........................................uagcaccaucugaaauG......   1       1       seq
.........................................uagcaccaucugaaaucg.....   235     0       seq
.........................................uagcaccauUugaaaucg.....   29      1       seq
.........................................uagcaccaucugaaaucgg....   1381    0       seq
.........................................uagcaccauUugaaaucgg....   115     1       seq
.........................................uagcaccauUugaaaucggu...   649     1       seq
.........................................uagcaccaucugaaaucggu...   5006    0       seq
.........................................uagcaccauUugaaaucgguu..   2668    1       seq
.........................................uagcaccaucugaaaucgguu..   57673   0       seq
.........................................uagcaccauUugaaaucgguua.   758     1       seq
.........................................uagcaccaucugaaaucgguua.   15626   0       seq
.........................................uagcaccauUugaaaucgguuau   56      1       seq
.........................................uagcaccaucugaaaucgguuau   1387    0       seq
..........................................agcaccauUugaaaucggu...   1       1       seq
..........................................agcaccaucugaaaucggu...   5       0       seq
..........................................agcaccauUugaaaucgguu..   1       1       seq
..........................................agcaccaucugaaaucgguu..   50      0       seq
..........................................agcaccaucugaaaucgguua.   13      0       seq
..........................................agcaccauUugaaaucgguua.   1       1       seq
...........................................gcaccaucugaaaucggu...   2       0       seq
...........................................gcaccaucugaaaucgguu..   5       0       seq
...........................................gcaccaucugaaaucgguua.   1       0       seq
............................................caccauUugaaaucgguua.   1       1       seq
..............................................ccaucugaaaucgguua.   1       0       seq
```
